# Promoting physical activity and health literacy: study protocol for a longitudinal, mixed methods evaluation of a cross-provider workplace-related intervention in Germany (The AtRisk study)

**DOI:** 10.1186/s12889-016-3284-6

**Published:** 2016-07-22

**Authors:** Andrea Schaller, Lea Dejonghe, Adrienne Alayli-Goebbels, Bianca Biallas, Ingo Froboese

**Affiliations:** IST University of Applied Sciences, Erkrather Str. 220 a-c, Düsseldorf, 40233 Germany; Institute of Health Promotion and Clinical Movement Science, German Sport University Cologne, Am Sportpark Muengersdorf 6, Cologne, 50933 Germany; Institute of Health Economics and Clinical Epidemiology (IGKE), University of Cologne, Gleuelerstr. 176-178, Cologne, 50935 Germany; Center for Health and Physical Activity, German Sport University Cologne, Am Sportpark Muengersdorf 6, Cologne, 50933 Germany

**Keywords:** Health promotion, Cross-provider, Workplace, Physical activity, Health literacy

## Abstract

**Background:**

Physical activity and health literacy are topics of utmost importance in the prevention of chronic diseases. The present article describes the study protocol for evaluating a cross-provider workplace-related intervention promoting physical activity and health literacy.

**Methods:**

The RE-AIM Framework will be the conceptual framework of the AtRisk study. A controlled natural experiment and a qualitative study will be conducted. The cross-provider intervention is based on the cooperation of the German Pension Fund Rhineland and cooperating German Statutory Health Insurances. It combines two components: a behavior-oriented lifestyle intervention and the assignment of a health coach. The single-provider intervention only includes the behavior-oriented lifestyle intervention.

The quantitative study (natural experiment) encompasses three measuring points (T0 = start of the behavior-oriented lifestyle intervention (baseline); T1 = end of the behavior-oriented lifestyle intervention (16 weeks); T2 = 6 month follow-up) and will compare the effectiveness of the cross-provider workplace-related intervention compared with the single provider intervention. Participants are employees with health related risk factors. ANCOVA will be used to evaluate the effect of the intervention on the outcome variables leisure time physical (primary outcome) activity and health literacy (secondary outcome). The qualitative study comprises semi-structured interviews, systematic field notes of stakeholder meetings and document analyses.

**Discussion:**

The AtRisk study will contribute towards the claim for cross-provider interventions and workplace-related approaches described in the new Preventive Health Care Act. The results of this study will inform providers, payers and policy makers about the effectiveness of a cross-provider workplace-related lifestyle intervention compared to a single-provider intervention. Beyond, the study will identify challenges for implementing cross-provider preventive interventions. With respect to the sustainability of preventive interventions the AtRisk study will give insight in the expectations and needs on health coaching from the perspective of different stakeholders.

**Trial registration:**

DRKS00010693.

## Background

Chronic diseases constitute the main cause of mortality and morbidity in the European Union [1]. Around 50 % of the populations in the age of 30 to 49 years suffer from one or more chronic diseases [2]. The demands on workers’ health have increased substantially due to skilled worker shortage, globalization, prolongation of working lifetime and transition to flexible working conditions. Hence, the prevention of chronic diseases to ensure employability is of high individual and socioeconomic importance [3–8].

It is widely known that influencing health contributes to a person’s well-being, quality of life, health status, workability and performance [9]. Health coaching offers opportunities for an individualized and sustainable prevention, especially by interconnecting aspects of behavior-oriented and condition-oriented (e.g. workplace) aspects and facilitating the client’s learning process by using professional methods and techniques [10, 11]. Within health promotion physical activity and health literacy are topics of utmost importance. Evidence shows that sedentary behavior is associated with an increased risk of premature all-cause and cardiovascular disease mortality and elevated biomarkers of cardio metabolic risk, including waist circumference, blood glucose, systolic blood pressure, and serum triglycerides [12–14]. Health literacy is a core competence and a high level of health literacy is considered of substantial benefit for maintaining one’s health [15, 16]. Health literacy describes a person’s knowledge and competence to meet the complex demands of health in modern society [16]. Several studies have already demonstrated the relationship of low health literacy, poor health status [9, 17, 18] and higher health care costs [19, 20].

The new Preventive Health Care Act in Germany points out the importance of workplace-related health promotion and calls for cross-provider interventions, e.g. cooperation of the German Statutory Health Insurance and the German Pension Fund. However, experiences on the capabilities and challenges of a cross-provider intervention are lacking. The present article describes the research protocol for the evaluation of a cross-provider workplace-related intervention promoting physical activity and health literacy. The specific objectives include (i) the evaluation of the effectiveness of a cross-provider workplace-related lifestyle intervention compared to a single-provider intervention; (ii) gaining understanding of the implementation of the cross-provider workplace-related lifestyle intervention during the study period; (iii) the identification of facilitators and barriers in cross-provider collaboration; as well as (iv) the identification of different stakeholders’ expectations and demands regarding health coaching.

## Methods/design

It is assumed that the cross-provider workplace-related intervention improves leisure time physical activity and health literacy compared to the single-provider intervention in persons who are at risk for chronic diseases.

### Research design and conceptual framework

The design and research questions of the AtRisk study were guided by the RE-AIM Framework [21]. The RE-AIM Framework is based on system-based and socio-ecological thinking and enables an overall assessment of the impact of the intervention. It has been specifically developed to facilitate the evaluation of interventions in real-world settings. The model identifies five evaluation dimensions: the *Reach,* the *Efficacy or Effectiveness*, the *Adoption*, the *Implementation* and the *Maintenance* of a public health intervention. The five RE-AIM dimensions occur at different levels, e.g. the individual, the organizational or the community level. *Reach* is defined as an individual-level measure of participation and refers to the percentage and risk characteristics of the participants. *Efficacy or Effectiveness* refers to the impact of an intervention on important intervention-specific outcomes, including potential negative effects, quality of life, and economic outcomes. *Adoption* refers to the proportion and representativeness of settings, e.g., such as worksites, health providers and/or payers, that adopt a given program or intervention. The *Implementation* refers to the extent to which a program is delivered as intended. At the setting level, implementation refers to the intervention providers’ fidelity to the various elements of an intervention’s protocol, including consistency of the delivery as intended and the time and cost of the intervention. Finally, the dimension *Maintenance* refers to the extent to which a program becomes institutionalized or part of the routine organizational practices and policies. Beyond, maintenance also refers to the individual level and has been defined as the long-term effects of a program on outcomes after six or more months after the most recent intervention contact [21]. Table 1 provides a description of the research questions for each of the five RE-AIM dimensions. The research questions will be answered by means of a mixed methods design combining quantitative and qualitative data sources. Quantitative data regarding intervention effectiveness will be collected using various questionnaires as part of a natural experiment. Qualitative data will be collected by means of supplementary interviews, informal field notes and document studies.

**Table 1:** Research questions and analytic approaches

Research question	Analytic approach
Reach	
1. How many persons participate in the intervention? 2. By what means were the participants recruited?	Quantitative study
	Qualitative study
Effectiveness	
1. Is there a difference in leisure time physical activity between the cross-provider intervention and the control intervention? 2. Is there a difference in health literacy between the intervention and the control intervention?	Quantitative study
	Quantitative study
Adoption	
1. How many providers offer the cross-provider intervention? 2. How many companies cooperate in the cross-provider intervention? 3. How many statutory social insurers cooperate in the cross-provider intervention?	Qualitative study
	Qualitative study
	Qualitative study
Implementation	
1. Is the intervention conducted according to the manual? 2. What are the facilitators and barriers for the cross-provider intervention? 3. What are the different stakeholders’ expectations and needs regarding health coaching in a work-related setting? 4. What are costs associated with implementation for the different stakeholders?	Qualitative study
	Qualitative study
	Qualitative study
	Qualitative study
Maintenance	
1. Is there a difference in leisure time physical activity between the cross-provider intervention and the control intervention at six month follow-up? 2. Is there a difference in health literacy between the intervention and the control intervention at six month follow-up? 3. What structures resources were built in companies, providers and social insurers to implement the cross-provider intervention in daily routine?	Quantitative study
	Quantitative study
	Qualitative study

### The cross-provider intervention

**Fig. 1:**
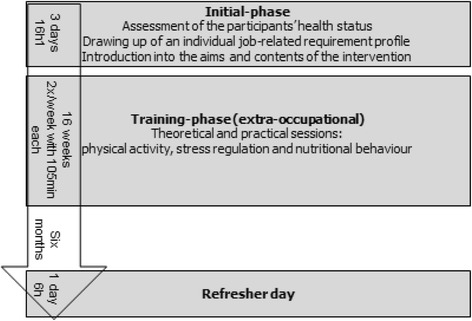
The behavior-related intervention of the German Pension Fund Rhineland (component A)
  The *initial-phase* comprises three days (or 16 h) and includes the assessment of the participants’ health status, the drawing up of an individual job-related requirement profile and the introduction into the aims and contents of the complete intervention and the subsequent training phase. The extra-occupational *training phase* is a behaviour-related preventive intervention comprising 32 trainings, 105 min each. The trainings are conducted twice a week over the duration of 16 weeks. The training phase combines theoretical and practical approaches and aims at a health-enhancing lifestyle change. The focus areas are physical activity, stress regulation and nutritional behaviour. The manual describes 15 theoretical sessions, 45 min each, spanning the subject areas of nutrition, physical activity, resilience and stress regulation. The 17 practical sessions comprise endurance training, coordination training, strengthening training with small devices and workplace related training sessions (45 min per session). In addition, medical training therapy is conducted at each of the 32 trainings (32 sessions, 60 min).
  Six months after the end of the training phase, the *refresher day* aims at the reflection of the participants regarding the lifestyle changes in daily routine. Individual barriers and facilitators are discussed with the group and the multiprofessional team.
  The maximum group size is 15 participants. To ensure the structure quality and the process quality of the behaviour-related preventive intervention is conducted by a multiprofessional team (sport scientists, psychologist, nutrition consultant) in an authorized ambulatory rehabilitation centre. To ensure the standardization of the preventive service, the content and structure of the intervention is based on a guideline and described explicitly in part two of *Präventionsleistungen der Deutschen Rentenversicherung Rheinland. Ein konzeptioneller Leitfaden* ([22]).
B) Personalized health coaching (optional)
  The cooperating statutory health insurances are financing the health coach. The health coach supports the participant during the entire period of the behavior-related lifestyle intervention (see A) and explicitly gives individual support between the end of the training phase and the refresher day (self-guided exercise). Health coaching focuses education, coaching and guidance of the participant. Health coaching is conducted by the means of face-to-face contact, telephone and e-mail contact. The superior aim of health coaching is bridging the interface between the behavior-related intervention (see A) and the workplace (see C). Beyond, health literacy and self-efficacy of the participant is supported. Frequency and content of health coaching varies according to the phase of the behavior-related intervention (see A) and the individual needs of the participant. For more information on the work of the health coach see *Präventionsleistungen der Deutschen Rentenversicherung Rheinland. Ein konzeptioneller Leitfaden* ([22]).

The cross-provider intervention is based on the cooperation of the German Pension Fund Rhineland and cooperating German Statutory Health Insurances. It incorporates the cooperating companies and combines two components: A) a behavior-oriented lifestyle intervention (for all participants) and B) assignment of a health coach (optional). The following components are offered within the comprehensive cross-provider health prevention service:

A) Standardized behavior-oriented lifestyle intervention (all participants)
  The German Pension Fund Rhineland finances a lifestyle intervention that comprises three phases: the initial-phase (three days), the training-phase (16 weeks) and a refresher day after 6 month of self-guided exercise (one day). The intervention is provided in four authorized ambulatory rehabilitation centres placed in North Rhine Westphalia, Germany (Fig. 1).

### Quantitative study

The quantitative study of AtRisk is a non-randomised controlled trial. The controlled trial made use of a natural experiment in which the implementation and delivery of the intervention was not manipulated by researchers. Instead, the allocation to one of the two possible conditions compared in this study (cross-provider intervention: A) and B); single-provider intervention: A)) is given according to the real world condition. If the statutory health insurance of the participant signed cooperation with German Pension Fund Rhineland in regard to preventive health services, the participant was allocated to the cross-provider intervention. Otherwise, the participant was allocated to the control group (single-provider intervention).

**Fig. 2:**
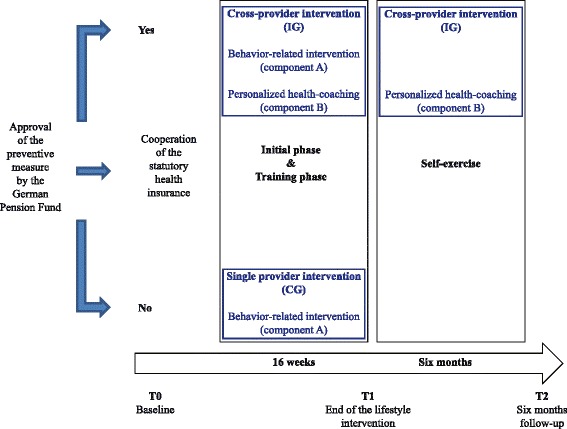
Allocation and study design

The AtRisk study compares the cross-provider intervention with a single provider intervention and encompasses three measuring points: T0 = start of the behavior-oriented lifestyle intervention (baseline); T1 = end of the behavior-oriented lifestyle intervention; T2 = 6 month follow-up (see Fig. 2).

The study was approved by the German Sport University Cologne Ethics Committee (reference number: 93/2015) and registered in the German Clinical Trials Register (ID: DRKS00010693). Written informed consent is taken from each participant. The recruitment of participants started in July 2016 and will be completed in August 2017. Six months follow-up (T2) will be completed in January 2018.

### Participants

The study population comprises employees with health related risk factors. Eligible participants were invited from the company doctor to participate in the behavior-related intervention (component A). The company doctor conducts the medical entry examination and the participant makes an application for the preventive measure at the German Pension Fund (§ 31 Social Code Book VI). The corresponding forms for the participant (G0180 and G0185) and the medical report of the company doctor (G0190) can be downloaded [23]. The application is approved by the German Pension Fund Rhineland. Eligibility criteria for participating in the present study is (1) a formally approved application for a preventive health service by the German Pension Fund, (2) age 18 to 65 years, (3) First health impairments (of the musculoskeletal system, internal organs or mental impairments) (4) written informed consent to participate in the study. Exclusion criteria are: (1) The indication for a rehabilitative treatment; (2) the need for acute care; (4) lack of understanding the German language.

### Outcome measurements

The participants answer a questionnaire on physical activity, health literacy, health-related quality of life and sociodemographic variables. The baseline questionnaire (T0) is answered at the beginning of the initial phase. The questionnaire at the end of the training phase (T1) and the 6 month follow-up questionnaire (T2) are collected using a postal questionnaire.

We chose leisure physical activity (MET^1^-min/week) as our primary outcome. Physical activity was operationalized by the Global Physical Activity Questionnaire [3, 24], which collects information on both physical activity during a typical week within three settings (workplace, transport and leisure time) as well as on sedentary behaviour. Activity specific scores are summed to give the total MET-min/week. Thereby, each minute of vigorous physical activity is multiplied by 8 METs and each minute of moderate physical activity by 4 METs. Bull et al. (2009) showed a moderate to strong positive relationship of the Global Physical Activity Questionnaire with the International Physical Activity Questionnaire (concurrent validity: Spearman’s rho 0.45–0.65), and the reliability was of moderate to substantial strength (kappa 0.67 to 0.73; Spearman’s rho 0.67 to 0.81) [3]. Compared to the accelerometer data, the Global Physical Activity Questionnaire provided low-to-moderate validity and generally acceptable evidence of reliability [25].

**Fig. 3:**
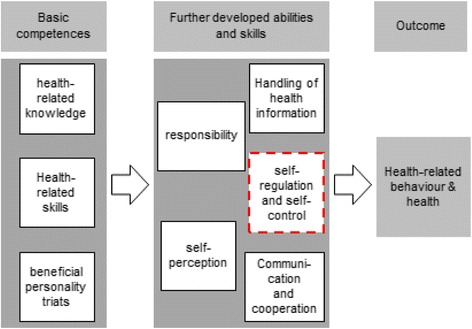
Structure model: further developed abilities and skills of health literacy ({Lenartz 2012 #4}, p. 120)

The secondary outcome measurement of the AtRisk study is health literacy. We assessed health literacy by the questionnaire from Lenartz [15] that is based on the definition of health literacy, representing “[…] the cognitive and social skills which determine the motivation and ability of individuals to gain access to, understand and use information in ways which promote and maintain good health.” ([26], p. 357). Lenartz (2012) developed a structure model explaining health behavior and health through the influence of individual basic competences (health-related knowledge, health-related skills and beneficial personality triats) and further developed abilities and skills. Based on this model, the health literacy scale from Lenartz comprises 30 items assessing six abilities and skills: self-regulation (5 items), self-control (5 items), self-perception (5 items) and responsibility (5 items), communication and cooperation (5 items) and the handling of health information (5 items) [15]. Each item has four response options”not correct at all”, “rather not correct”, “rather correct”, “correct” (Fig. 3).

Additionally, the domain „Actively managing my health“of the Health Literacy Questionnaire (HLQ) is used [27]. This domain contains five items (“I spend quite a lot of time actively managing my health”; “I make plans for what I need to do to be healthy”; “Despite other things in my life, I make time to be healthy”; “I set my own goals about health and fitness”; “There are things that I do regularly to make myself more healthy”) measured by a four point likert-scale (strongly disagree, disagree, agree and strongly agree). The scale score is devised by summing the item scores and dividing by the number of items in the scale [27].

### Further variables

For obtaining person-related variables, age, sex, height, weight, education level, family status and health-related lifestyle were assessed by unstandardized questions. Beyond, health-related Quality (HRQoL) of life was measured using the EQ-5D-5 l questionnaire [28]. The EQ-5D is a standardized measure of health status and assesses five dimensions of HRQoL: mobility, self-care, daily activities, pain and discomfort, anxiety or depression. EQ-5D-5 L provides five levels for answering each dimension: “no problems”, “slight problems”, “moderate problems”, “severe problems” and “extreme problems”. Descriptive answers were converted into a single summary index [29]. Index value of health status range from ‘1’ (perfect health) to ‘0’ (death). Beyond, workability was assessed by the work ability index (WAI) [30, 31] employability was measured by the 3-item subjective prognosis of gainful employment (SPE-scale) [32–34]. Overall, the following instruments are used (Table 2).

**Table 2:** Summary of measures

	Instrument	Time of measurement^a^
Primary outcome measure		
Physical Activity	GPAQ Questionnaire ([3, 24])	T0, T1, T2
Secondary outcome measure		
Health literacy	Health literacy scale ([15])	T0, T1, T2
	HLQ Subscale „Actively managing my health“([27])	
Person-related variables		
Age, sex, height, weight,	Unstandardized questionnaire	T0
Education level, family status	Unstandardized questionnaire	T0
Health-related lifestyle	Unstandardized questionnaire	T0, T1, T2
Health Related Quality of Life	EQ-5D-5 L ([28])	T0, T1, T2
Work-related variables		
Workability	Work ability questionnaire ([30, 31])	T0, T1, T2
Employability	subjective prognosis of gainful employment (SPE-scale) (Mittag 2003 #57}; [33, 34])	T0, T1, T2

^a^ T0 = initial phase (baseline); T1 = End of training-phase; T2 = six month follow-up

### Statistical analysis

Descriptive statistics will be used to describe the baseline characteristics of study population and to explore the distributions of the variables at the individual level. Depending on the distributions of the quantitative variables parametric and non-parametric tests are used to compare the intervention and the control group at baseline.

The two study groups will be compared using ANCOVA, with the outcome variables leisure time physical (primary outcome) activity and health literacy (secondary outcome), at 6 month follow-up set as the dependent variable [35]. Baseline values and the rehabilitation center will be used as a covariate to control for imbalance between control and intervention groups at baseline.

Standardized mean-difference effect size will be calculated by using the means of the two groups, mean-square error and the correlation between the covariate and dependent variable.

Intervention outcomes will be evaluated in relation to the base case data but also to the intention to treat principle. The base-case analysis will be performed using data restricted to those patients who replied to the postal 6 month follow-up questionnaire. The intention-to-treat analysis (ITT_LOCF_) will be performed assuming “last observation carried forward” since the effectiveness of physical activity promotion is considered controversial [36–38]: if 6 month follow-up data (T2) were missing, it was assumed that the physical activity data were the same as at baseline (T0). The statistical analyses will be conducted independently of the research team. The data will be analyzed using the statistical software IBM Statistics 23.0.

The calculated total sample size is 237 (ANCOVA: Fixed effects, main effects and interactions; Input: effect size f = 0.25 with 80 % power at p < 0.05; number of groups = 8 (group = 2; ambulatory rehabilitation center = 4); numerator df = 7; number of covariates = 2 (age; baseline value)). Therefore, it is estimated that a sample size of 119 participants per study arm would be required to detect a medium effect between the cross-provider intervention and the single-provider intervention. To allow for missing data and loss to follow up, we aim to recruit 150 participants per study arm.

### Qualitative study

The qualitative study comprises semi-structured interviews, systematic field notes of stakeholder meetings and document analyses. Semi-structured interviews and field notes will be conducted to explore the questions in regard to the RE-AIM dimensions i*mplementation* (“What are the facilitators and barriers for the cross-provider intervention?”; “Is the intervention conducted according to the manual?”; “What are the different stakeholders’ expectations and needs regarding health coaching in a work-related setting?”; “What are costs associated with implementation for the different stakeholders?”) and *maintenance* (“What structures resources were built in companies, providers and social insurers to implement the cross-provider intervention in daily routine?”) (see Table 1). The different stakeholders involved in the intervention (German Pension Fund Rhineland, cooperating statutory health insurers, providers, health-coaches, participants, company doctors and company management) will participate in the semi structured interviews.

In regard to health-coaching the semi-structured interviews encompass the perspectives of different stakeholders. Amongst others, the aim is to get information on tasks and objectives of health coaching, the necessary qualification, skills and competences of a health coach as well as the working methods and main topics in health coaching. The interview partners will be interviewed personally once, using semi-structured qualitative interviews. Two researchers lead through the semi-structured interviews: one will lead the conversation and the other one will do the documentation. If the participants agree, the interviews will be audio recorded. All semi-structured interviews will be in German. After the interviews are completed, transcriptions will be done according to the rules of Kuckartz [39]. Transcriptions will be double-checked.

The transcripts will be analysed according to the structuring content analysis by Mayring [40], which is similar to the framework approach [41]. For the analysis a qualitative research specialist at German Sport University Cologne, Germany will use the software MAXQDA 12 in order to code the information and look for relevant trends and themes within the data. Common themes and issues will be grouped together in main- and sub-categories and frequencies will be noted in order to highlight key issues. Though this is a qualitative study, the frequencies will be reported. However, direct quotations will be pulled from the session reports when they are illustrative of the issues. Overall, around 15 interviews will be included in this study, approximately two interviews per stakeholder group. Beyond, field notes of protocols of conversations with stakeholders (German Pension Fund Rhineland, statutory health insurances, health coaches, therapists and managers of ambulatory rehabilitation centers) and documents will be considered to gain insight into the dimension *adoption* (“How many providers offer the cross-provider intervention?”; “How many companies cooperate in the cross-provider intervention?”; “How many statutory social insurers cooperate in the cross-provider intervention?”) and i*mplementation* (“Is the intervention conducted according to the manual?”).

## Discussion

The study presented is designed to evaluate the effectiveness and the challenges of a workplace-related cross-provider intervention. Beyond, the expectations and needs on health coaching are of particular interest.

The study took as its starting point the new Preventive Health Care Act in Germany and its claim on cross-provider preventive measures [42, 43]. The second starting point of the study was significance and also the challenges of workplace-related health promotion. Despite the importance of workplace-related health promotion, the feasibility and the effectiveness are widely debated [24, 44]. On the one hand, the workplace is suitable reaching workers with health-risks for preventive measures [45–47]. On the other hand, participation rates in health promotion interventions at the workplace are low [25]. Overall, studies relating to workplace interventions are of high heterogeneity concerning interventions as well as population groups and therefore rarely comparable [48]. Nevertheless, study results support the superiority of multimodal interventions including ergonomic, behavior-related and workplace-related aspects compared to single interventions [49]. Workplace-related interventions should be multimodal and tailored to the needs and the preventive competence of both, the individuals and the organizations [50].

In regard to practical implications results may be relevant because of the clear orientation in employees with health-related risk factors. Since most studies focus mixed populations and do not differentiate interventions aiming at populations at risk and healthy persons [51] it is essential not only to develop but also to evaluate interventions with the ability to reach employees focusing on those who need it most [25, 51]. Hence, especially persons with health related risk factors (e.g. physical inactivity), people with a low socioeconomic status and men show a low rate of preventive activities [52, 53]. Beyond, the evaluation of the effectiveness of health coaching in regard to the sustainability of the preventive lifestyle measure is of high practical relevance. The study contributes to the professionalization in the field of health coaching not only by the evaluation of its effectiveness but also by the semi-structured interviews with different stakeholders providing a more profound understanding of the potential, challenges and qualification needs in health coaching.

There are several challenges in conducting this study. First, the present evaluation is performed in a real-life setting. This is a strength, on the one hand, because this provides a realistic estimate of effectiveness. On the other hand, several methodological challenges arise. Workplace-related interventions as well as studies promoting physical activity are fraught with methodological difficulties. Evidence development is considered a big challenge in prevention and health promotion [54–57]. Measures in health promotion are mainly complex interventions in complex socio-ecological systems [58] and several dimensions of complexity exist, for example the range of possible outcomes, the outcome variability in the target population and the number of elements in the intervention package itself [59]. Irrespective of the mode of intervention, evaluation of the impact therefore underlies high methodological standards and needs to consider three perspectives: multiplicity, context sensitivity and complexity as a reference point [56]. Although for many decades randomized controlled experiments have dominated the impact assessment of social or health programs, there are many arguments that stress the artificiality of these approaches as well as the lack of useful information produced [60, 61]. The World Health Organization even concluded that “the use of randomized control trials to evaluate health promotion initiatives is, in most cases, inappropriate, misleading and unnecessarily expensive” ([62], p. 5).

Realistic evaluation considers the complexity of health interventions and it may help to meet the challenges of evaluation in health promotion aiming at finding out how a program works, for whom and under what circumstances. The RE-AIM Framework provides a useful template to guide the design and implementation of a cross-provider intervention as it allows concurrent evaluation of dimensions considered relevant to ‘real world’ implementation.

The use of mixed methods within the research project enables a more complete picture of the intervention, its effectiveness and finding out how the intervention works, for whom and under what circumstances [63].

## Footnotes

1 The metabolic equivalent (MET) is a physiological measure expressing the expended energy of physical activities. MET is defined as the ratio of the rate of energy consumption during a specific physical activity to a reference metabolic rate.
